# Autonomic Nervous System Activity During a Speech Task

**DOI:** 10.3389/fnins.2019.00406

**Published:** 2019-05-08

**Authors:** Naomi Dodo, Ryusaku Hashimoto

**Affiliations:** ^1^Department of Clinical Psychology, School of Psychological Science, Health Sciences University of Hokkaido, Tōbetsu, Japan; ^2^Department of Communication Disorders, School of Rehabilitation Sciences, Health Sciences, University of Hokkaido, Tōbetsu, Japan

**Keywords:** autonomic nervous system responses, speech, active coping, cardiac sympathetic index, cardiac vagal index, stress

## Abstract

Previous research has reported that different coping types (active or passive) are required depending on the stress-inducing task. The aim of this study was to examine the autonomic nervous response during speech tasks that require active coping, by using Lorenz plot analysis. Thirty-one university students participated in this study (*M* = 21.03 years, *SD* = 2.27). This study included 3 phases: (1) resting phase, (2) silent reading phase, and (3) reading aloud phase. Autonomic nervous system responses were recorded in each phase. We asked participants to evaluate their subjective states (arousal, valence, and mood) after the silent reading phase and the reading aloud phase. We observed that the cardiac sympathetic index (CSI) for the sympathetic nervous response was significantly higher during the reading aloud phase than during the silent reading phase. In contrast, the cardiac vagal index (CVI) for the parasympathetic nervous response was significantly higher during the reading aloud phase than during the resting phase. There were no significant differences between the resting phase and the silent reading phase in both cardiac sympathetic and CVIs. We also observed that the degree of arousal was significantly higher after the reading aloud phase than after the silent reading phase. Our findings indicate that the psychological load during silent reading is ineffective for activating the sympathetic nervous system. The sympathetic nervous response was activated in the reading aloud phase. Also, the parasympathetic nervous response in the reading aloud phase was activated compared with the resting phase. Reading aloud is necessary to adequately activate the parasympathetic nervous system by requiring participants to respire (i.e., expiration) more than during resting and silent reading tasks. The increase in the CVI likely stems from activating the parasympathetic nervous system during expiration. Although the speech task required participants to perform active coping, it was designed to activate both the sympathetic and parasympathetic nervous systems during expiration.

## Introduction

Both time-domain analysis and frequency-domain analysis are used as methods of analyzing autonomic nervous response. Billeci et al. (2018) and Dodo and Hashimoto (2015) recently used both methods of analysis. Frequency-domain analysis (spectral analysis) of short (≤5 min) interbeat interval (IBIs, or R–R intervals) time series typically yields 3 peaks: 0–0.04 Hz, very low frequency (VLF); 0.04–0.15 Hz, low frequency (LF); and 0.15–0.40 Hz, high frequency (HF). The HF component represents parasympathetic nervous system activity and is strongly influenced by respiratory sinus arrhythmia. VLF and LF are used as indicators of sympathetic nervous system activity. However, as noted by Sawada (1999) and Lahiri et al. (2008), the LF and VLF components are affected by both sympathetic and parasympathetic nervous system activities. Therefore, it is difficult to use spectrum analysis to independently measure sympathetic nervous system activity. Lorenz plot analysis is a type of time-domain analysis. Notably, Lorenz plot analysis allows parasympathetic and sympathetic nervous system activities to be measured separately (Toichi et al., 1997). Lorentz plot analysis uses the cardiac sympathetic index (CSI) as an indicator of sympathetic nervous system activity; it uses the cardiac vagal index (CVI) as an indicator of parasympathetic nervous system activity. Dodo and Hashimoto (2015) found that time-domain analysis (Lorenz plot analysis) is a useful method for examining autonomic nervous system activity during a cold presser test (CPT), whereas frequency-domain analysis (spectral analysis) of heart rate variability (HRV) is not.

Obrist (1981) suggested that different coping types (active or passive) are required depending on the stress-inducing task. With active coping, sympathetic nervous system activity increases and parasympathetic nervous system activity decreases from baseline. During passive coping, sympathetic nervous system activity decreases and parasympathetic nervous system increases from baseline. Allen et al. (2007) reported results of a mental arithmetic task that requires active coping using Lorenz plot analysis. In the mental arithmetic task, the CSI increased and the CVI did not change, compared to baseline. Dodo and Hashimoto (2015, 2017) conducted CPT studies that required passive coping. In CPT, the CSI did not change and the CVI increased, compared to baseline. Indexes activated during tasks that required active or passive coping were consistent with those identified by Obrist; active coping activated CSI, whereas passive coping activated CVI.

Other tasks that require active coping include speech and mental arithmetic tasks. Most studies of HRV during speech tasks use frequency-domain analysis (spectral analysis; for review, see Bernardi et al., 2001). However, respiratory patterns change with utterance during speech. When analyzing HRV using spectral analysis, Sawada (1999) noted that, with changes in respiratory patterns, the values of both HF and LF components increase. Beda et al. (2007) compared tasks with and without utterance. They suggested that the use of spectral analysis to assess autonomic function during tasks with utterance was problematic.

Therefore, our study aimed to use Lorentz plot analysis (i.e., independent analysis of the sympathetic and parasympathetic nervous systems) to examine influence on the autonomic nervous system during tasks with utterance.

## Materials and Methods

### Participants

We selected 40 university students to participate in this study. For participating in the experiment, we paid approximately 9 US dollars (1,000 Japanese yen) to each participant, and we provided all students with written informed consent forms before participation. We asked them to refrain from eating or drinking anything other than water for at least 2 h before arriving at the laboratory. We excluded four volunteers because they were smokers or were taking medications. Because smoking and medications affect the autonomic nervous system, these were not permitted. We also excluded five volunteers due to electrocardiogram (ECG) artifacts. Thus, we included 31 participants (10 male and 21 female; age range: 19–29 years; mean age: 21.03 years; SD age: 2.17).

### Procedure

#### Stimuli

We prepared several stories that cited the famous classical essay “Tsurezuregusa” (“Essays in Idleness”) by Yoshida Kenkō (1330–1332). Words and expressions used in classical literature are rarely used in modern literature. Therefore, participants could not easily read the stories, as they were difficult to comprehend. We selected four of the less familiar stories from the essay. We divided each story into 12 texts. We presented the 12 texts on a display at a constant pace. Each text was presented every 13 s. The intertext interval was 2 s.

#### Experiment

The study was composed of three phases: (1) resting phase (R phase), (2) silent reading phase (SR phase), and (3) reading aloud phase (RA phase). Each phase lasted 3 min. Participants were seated and maintained their posture throughout the experiment. In the R phase, they watched a silent movie. In the SR phase, they were shown text on the display and read it in silence. During the RA phase, we asked them to read the text presented on the screen with clear enunciation. Based on the findings of Piferi et al. (2000), we streamed a monochrome silent movie in the R phase. The content of the movie was summer flower scenery that did not change the psychological state of participants. We gave the following instructions to participants prior to the movie: “Please sit back in the chair. Please watch the movie in a relaxed posture without moving.”

To prevent the autonomic nervous system from being affected by the content of the presented story, we randomly showed one of the four stories in each phase. We monitored participants’ faces using a video camera placed in front of them and observed their commitment to the task. Furthermore, we recorded any comments they made with their consent.

We asked participants to evaluate their subjective states (degrees of arousal and valence) and mood after the silent reading and reading aloud phases. After the RA phase, all participants evaluated their performance on the reading aloud task (self-evaluation).

#### Psychological Estimation

##### Subjective state

We queried participants’ degree of arousal and valence according to the dimensions of affect proposed by Russell et al. (1989). We asked participants to assess their subjective states (degrees of arousal and valence), using a seven-point Likert scale. For the degree of arousal, the median score (4) was neutral. The highest score represented low arousal (7), while the lowest score denoted high arousal (1). Regarding valence, the highest score denoted more positive affect (7), the lowest score denoted more negative affect (1), and the scale midpoint (4) represented neutral affect.

##### Mood

We asked participants to judge their subjective moods (happy, melancholy, feeling pleasure, sad, lonely, or satisfied) using a seven-point Likert scale (Sakaki, 2006). Each descriptor was scored from 1 (not applicable) to 7 (perfectly applicable). We applied reverse scoring for three descriptors (melancholy, sad, and lonely). We recorded the total score of the six descriptors as the mood score.

##### Self-evaluation

We directed participants to rate their performance (fluency) on the reading aloud task, using a seven-point Likert scale that ranged from poor (1) to excellent (7), while the midpoint (4) denoted average performance.

#### Autonomic Nervous Response

We recorded heart rate in all three phases. To assess HRV, we administered an ECG with three Ag–AgCl disposable electrodes (PSC-SC43m, Senstec Co., Ltd., Tokyo, Japan) arranged in a similar manner to that of a lead II configuration (i.e., two electrodes on the breastbone and one on the left lower abdomen). We digitized the ECG data using a 12 bit A/D converter at a sampling rate of 1 kHz (MaP222A, NIHONSANTEKU Co., Ltd., Osaka, Japan) and recorded data to a notebook computer (T60, IBM Japan, Ltd., Tokyo, Japan).

We evaluated HRV using Lorenz plot analysis (MaP1060, NIHONSANTEKU Co., Ltd., Osaka, Japan). We observed fluctuations of the IBI and transformed them into an ellipsoid distribution using the Lorenz plot. Following Toichi et al. (1997), a program (MaP1060) calculated the length of the longitudinal (L) and transverse (T) axes within the ellipsoid distribution. The CVI was calculated as a log10 (L × T) transformation, and the CSI was calculated as L/T (Toichi et al., 1997).

### Statistical Analyses

The data were analyzed using IBM SPSS, Version 25. The Shapiro–Wilk test was applied to evaluate whether the variables considered were normally distributed. If the data had normal distribution, we performed a paired *t*-test for psychological measures or a one-way repeated-measure analysis of variance (ANOVA) for autonomic nervous response with phases. If the data had a non-normal distribution, we performed the Wilcoxon signed-rank test for psychological measures or the Friedman test for autonomic nervous response among phases. We carried out *post hoc* analyses with Bonferroni correction.

## Results

The Shapiro**–**Wilk test was applied to evaluate whether the variables considered were normally distributed. The data were analyzed using IBM SPSS, Version 25.

### Psychological Measures

#### Subjective State

According to the results of the Shapiro–Wilk test, state had a non-normal distribution in the R, SR, and RA phases. Thus, we used the Wilcoxon signed-rank test for comparison of subjective state. Arousal scores were significantly higher after the SR phase (*M* = 4.26, *SD* = 1.26) than after the RA phase (*M* = 3.03, *SD* = 1.17) (*p* < 0.05). This result means that the degree of arousal was higher after the RA phase. Valence scores were not significantly different after the SR phase (*M* = 4.48, *SD* = 0.89), relative to those of the RA phase (*M* = 4.23, *SD* = 0.99; Table 1).

**Table 1:** Psychological estimation.

	After SR phase		After RA phase	
**Subjective State**		
Arousal	4.26	(1.26)	3.03	(1.17)
Valence	4.48	(0.89)	4.23	(0.99)
Mood	27.74	(4.88)	28.35	(5.90)


*Values for subjective state and mood scores are expressed as mean (SDs).*

#### Mood

According to the results of the Shapiro–Wilk test, mood had a non-normal distribution in the R, SR, and RA phases. Thus, we used the Wilcoxon signed-rank test for comparison of mood; notably, it did not significantly differ between the SR phase (*M* = 27.74, *SD* = 4.88) and the RA phase (Table 1).

#### Self-Evaluation

The mean self-evaluation score was 3.26 (*SD* = 1.41).

### Autonomic Nervous Response

**FIGURE 1:**
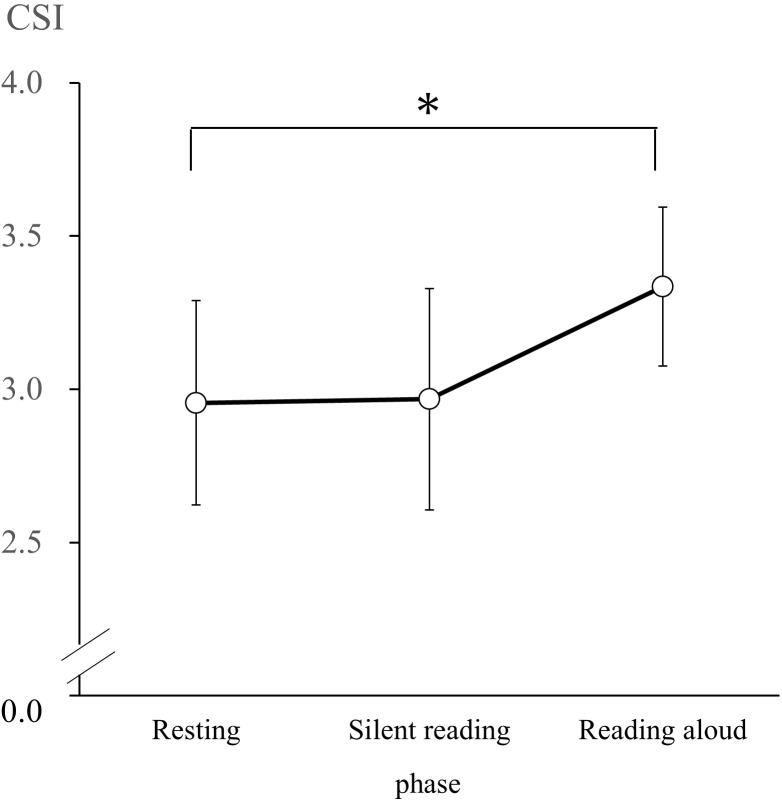
Cardiac sympathetic index (CSI) changes during the resting, silent reading, and reading aloud phases. Values are expressed as means and SDs. ^∗^*p* < 0.05.

**FIGURE 2:**
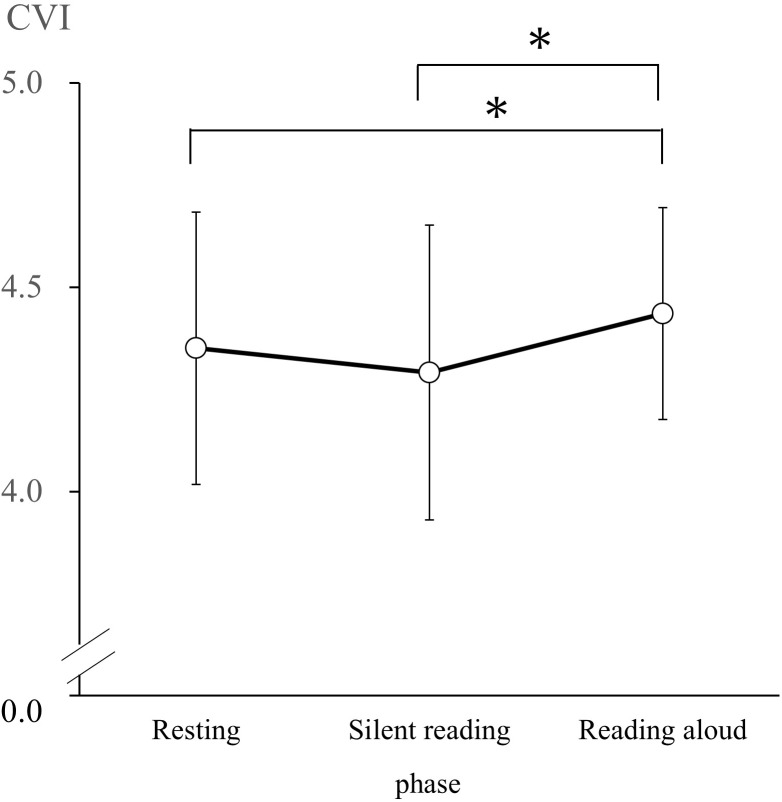
Cardiac vagal index (CVI) changes during the resting, silent reading, and reading aloud phases. Values are expressed as means and SDs. ^∗^*p* < 0.05.

According to the results of the Shapiro–Wilk test, CSI and CVI had normal distributions in the R, SR, and RA phases. Thus, we conducted a one-way repeated-measures ANOVA of CSI and CVI values with phase (resting, silent reading, and reading aloud) as the factor. We observed a significant main effect of the phase on CSI [*F*(2, 60) = 3.95, *p* < 0.05, $\eta_{p}^{2}$ = 0.12]. CSI values during the R phase (*M* = 2.96, *SD* = 0.91) were significantly lower than in the RA phase (*M* = 3.33, *SD* = 0.95; *d* = 0.40; Figure 1). We observed a significant main effect of phase on CVI [*F*(2, 60) = 7.40, *p* < 0.05, $\eta_{p}^{2}$ = 0.20]. CVI values in the R phase (*M* = 4.35, *SD* = 0.33) and the SR phase (*M* = 4.29, *SD* = 0.36) were significantly lower than those in the RA phase (*M* = 4.44, *SD* = 0.26; R versus RA: *d* = 0.30, SR versus RA: *d* = 0.48; Figure 2).

## Discussion

We observed that the degree of arousal was significantly higher after the RA phase than after the SR phase. Moreover, the sympathetic nervous response was significantly higher during the RA phase than during the SR phase, and the parasympathetic nervous response was significantly higher during the RA phase than during the R phase.

### Psychological Response

The degree of arousal was greater after the RA phase than after the SR phase. However, there was no difference in valence after the SR phase, nor after the RA phase. In contrast, because there is no significant difference between valence and moods, the emotional influence on the autonomic nervous system caused by the tasks is considered equivalent for the silent reading and reading aloud conditions. The self-evaluation scores were slightly lower than the midpoint (“average”) of the seven-category scale. Thus, participants evaluated their reading quality as below average. This signifies that the chosen text was challenging. According to the results of the psychological ratings, reading aloud produces greater psychological loading.

### Autonomic Nervous System

There was no significant difference between CSI values, which is an index sympathetic nervous system activity, during the resting and silent reading phases. However, it was found that CSI values significantly increase during the RA phase rather than in the SR phase. The CSI findings during the RA phase are consistent with an increase in the sympathetic nervous system due to tasks requiring active coping, as noted by Obrist (1981). The CSI did not increase more in the SR phase than during the R phase; this finding suggests that psychological loading in the SR phase is ineffective as an active coping task.

Obrist (1981) indicated that the parasympathetic nervous system activity decreases below baseline during tasks that require active coping. However, our data show that the CVI, a measure of the parasympathetic nervous system, rises significantly more during the RA phase than in the R phase. The reading aloud task requires vocalization and causes participants to respire (expirate) more than in the resting and silent reading tasks. Increases in the CVI are considered to result from activating the parasympathetic nervous system by said expiration. Although the speech task required participants to perform active coping, it also activated both the sympathetic and parasympathetic nervous systems during expiration.

Allen et al. (2007) utilized Lorenz plot analysis with a mental arithmetic task that required active coping; they found that the CSI increased during the arithmetic task, which was consistent with our findings in CSI during the RA phase. We speculate that the increase in CSI (i.e., the sympathetic nervous system index) arose from active engagement in the task among participants. Allen et al. (2007) also reported that the CVI did not change during the mental arithmetic task. However, we found that the CVI increased during the RA phase. In the RA phase, respiratory pattern changed with utterance, but not with the mental arithmetic task. The difference in respiratory pattern may have led to the difference in CVI between RA and mental arithmetic tasks. We considered that the increase in CVI (i.e., the parasympathetic nervous system index) during the RA phase resulted from changes in respiratory pattern during speech.

### Limitations

The main purpose of this study was to examine influence on the autonomic nervous system during a reading task with utterance. However, we did not confirm if the participants were reading with effort or not reading at all during the SR phase. For example, a memory task is a method for confirming the engagement of the participants in reading. Adding a memory task to the reading tasks would change this to a dual task, which is a higher psychological load. Thus, we would not be able to clearly discern the effect of utterance alone. In the present study, we did not add a memory task during silent reading or reading aloud. We monitored participants’ faces using a video camera placed in front of them; thus, we observed their commitment to the task. No participants closed their eyes except to blink during the SR phase.

We did not perform the SR and RA phases in random order. The outcome of the RA phase may have been contaminated by habituation. However, arousal during the RA phase was significantly higher than in the SR phase. This suggests that habituation to the reading task did not affect the outcomes of the RA phase.

## Conclusion

For speech tasks requiring active coping, we separately analyzed sympathetic and parasympathetic nervous system activity, using Lorenz plot analysis. Our results suggest that each effect on the autonomic nervous system is evoked by two different behaviors: one behavior was the action of reading aloud, which required active coping and led to the activation of the sympathetic nerve system. The other behavior was the action of speech with utterance; respiratory pattern changed during speech, and this change led to the activation of the parasympathetic nervous system. When evaluating the activity of the autonomic nervous system in tasks associated with utterance, Lorenz plot analysis is recommended.

## Data Availability

The datasets generated for this study are available on request to the corresponding author.

## Ethics Statement

This study was carried out in accordance with the recommendations of the Psychological Science Ethics Review Committee guidelines, the Ethics Committee of Health Sciences University of Hokkaido. The protocol was approved by the Ethics Committee of Health Sciences University of Hokkaido. All subjects gave written informed consent, in accordance with the Declaration of Helsinki.

## Author Contributions

ND contributed to the design of the experiment, performance of the experiment, data analysis, and writing of the manuscript. RH contributed to the design of the experiment, performance of the experiment, and writing of the manuscript.

## Conflict of Interest Statement

The authors declare that the research was conducted in the absence of any commercial or financial relationships that could be construed as a potential conflict of interest.

## Abbreviations

CSI: cardiac sympathetic index
CVI: cardiac vagal index
HF: high frequency (0.15–0.40 Hz)
LF: low frequency (0.04–0.15 Hz)
R phase: resting phase
RA phase: reading aloud phase
SR phase: silent reading phase
VLF: very low frequency (0–0.04 Hz)
